# Anti-Obesity Effects of Tocotrienols and Bran in High-Fat Diet-Treated Mice

**DOI:** 10.3390/nu11040830

**Published:** 2019-04-12

**Authors:** Koji Fukui, Masashi Shirai, Takeyuki Ninuma, Yugo Kato

**Affiliations:** Molecular Cell Biology Laboratory, Systems Engineering and Science, Graduate School of Engineering and Science, Shibaura Institute of Technology, Fukasaku 307, Minuma-ku, Saitama 337-8570, Japan; mf15041@shibaura-it.ac.jp (M.S.); mf18063@shibaura-it.ac.jp (T.N.); nb19102@shibaura-it.ac.jp (Y.K.)

**Keywords:** tocotrienols, bran, high-fat, anti-obesity, oxidative stress

## Abstract

Obesity is a serious public health issue in developed countries, and is known to increase the risk of several diseases such as diabetes, cardiovascular events and arteriosclerosis. These phenomena are closely correlated with oxidative damage. Recently, several lines of evidence have demonstrated that neurodegenerative diseases such as Alzheimer’s and Parkinson’s are also related to oxidative damage. To clarify the relationship between obesity and oxidative brain injury, we investigated brain antioxidant networks in high-fat (HF) diet-treated mice in the presence or absence of tocotrienols (T3s) and bran. Co-treatment with T3s and bran significantly inhibited bodyweight gain in HF diet-treated mice. Serum and cortex T3 levels, and brain antioxidant enzyme activities and protein expressions did not differ among the groups except for SOD protein expression in the cerebellum. Brain p-mTOR and p-Akt protein expressions, which are related to autophagy, did not differ among the groups. These results indicate that treatment with T3s for eight weeks had showed an anti-obesity effect in HF diet-treated mice. However, significant alterations in T3 levels were not observed in the serum and brain of mice.

## 1. Introduction

Obesity is a serious global issue [1,2] that is characterized by a high body-mass index (BMI, weight in kilograms divided by the square of the height in meters), and its incidence is gradually increasing in Japan. The most important issue related to obesity is its contribution to the onset of serious diseases such as cardiovascular disease [3], type 2 diabetes mellitus [4], and hypertension [5]. To prevent the onset of these diseases, it is important to maintain an appropriate body weight by controlling food intake and physical exercise. Several lines of evidence have demonstrated that calorie restriction has beneficial effects on health and lifespan extension [6,7,8]. The majority of current studies on calorie restriction and lifespan extension employ rodents [6] and non-human primates [9], and the results indicated that sirtuin 1 expression [10], known as the “longevity gene”, and down-regulation of metabolic rate [11] play a role. Reduced oxygen consumption is a key factor in calorie restriction-related lifespan extension [12]; in other words, obesity patients may exhibit high oxygen consumption, possibly shortening their lifespan. It is well known that oxidative stress is a factor in obesity [10]. In fact, reactive oxygen species (ROS)-derived oxidative damage accelerates pathological aging, known as “the free radical theory of aging” [13], and increases the risk of the development and progression of severe diseases, such as neurodegenerative disorders [14,15]. The aging process is a universal and unavoidable phenomenon. However, the detailed relationship between ROS-derived oxidative damage in the brain and obesity has not been elucidated.

Tocotrienols (T3s) are natural lipophilic vitamin E (VE) homologues that function as antioxidants [16]. T3s are present in a variety of foods including annatto, palm, whole wheat, rice bran, etc. Recently, other beneficial functions of T3s have been reported, such as the neuroprotective effect of α- and γ-T3 [17], the inhibitory effect of γ-T3 on hydroxymethylglutaryl-CoA (HMG-CoA) reductase [18] and the induction of apoptosis in cancer cells of a T3-rich fraction (TRF) extracted from palm oil [19]. Furthermore, there are reports that T3s such as γ-, δ-T3 and TRF have an anti-obesity effect [20,21,22]; however, the detailed mechanism of the T3s-related anti-obesity effect has not yet been elucidated due to paucity of evidence. Whole grain wheat, which contains the epidermis and germ, is commonly consumed in Western cultures. Recently, its potential in health promotion has garnered attention in Japan, and there has been a gradual increase in its consumption. Whole wheat contains fiber and minerals as well as large amounts of T3s compared to normal white flour. We hypothesized that treating obese mice with whole grain wheat should produce an anti-obesity effect due to the presence of T3s. In the present study, we treated high-fat (HF) diet-treated mice with bran or T3s and monitored the effect on body weight. We also investigated the relationship between obesity and brain oxidative damage by measuring antioxidant levels in the mouse brain.

## 2. Materials and Methods

### 2.1. Animals and Diets

All animal experiments were performed with the approval of the Animal Protection and Ethics Committee of the Shibaura Institute of Technology (Tokyo, Japan). Four-week-old normal male mice (wild-type C57BL/6) were obtained from Japan SLC, Inc. (Hamamatsu, Japan). Mice were randomly assigned to one of five dietary groups: control (Ctrl, *n* = 8), high fat (HF, *n* = 7), control + T3 (Ctrl + T3, *n* = 8), HF + T3 (*n* = 8), and HF + 5% bran (*n* = 8). HF model mice were generated by feeding a diet (#D12492; Research Diets Inc., New Brunswick, NJ, USA) containing 5.24 kcal/g, with 60% of the calories from fat, 20% from protein and 20% from carbohydrates to normal 4-week-old mice until 12 weeks of age. Control diets (CD) (#D12450J; Research Diets Inc.) containing 3.85 kcal/g, with 10% of the calories from fat, 20% from protein and 70% from carbohydrates were used for the control groups (Supplemental Table S1). Ten milligrams of a T3s mix (α, 33.2%, β, 4.2%, γ, 46.1%, δ, 15.0%), which was provided by Mitsubishi-Chemical Foods Corp. (Tokyo, Japan), was mixed with 100 g of CD and HF, respectively. Five grams of bran (Ebetsu Flour Milling Co., Ltd., Ebetsu, Japan) was mixed with 100 g of HF. The feeding period of these foods was the same as for HF. The mice were also fed the diets until 12 weeks of age. Animals were housed under conditions of a 12-h light/dark cycle and a temperature of 24 ± 2 °C. During the study, all animals were provided access to food and water ad libitum. Food intake and body weight were measured on a weekly basis. At the end of the feeding period, the mice were administered ether anesthesia and sacrificed by decapitation; the serum was collected and all organs were removed for analysis. All other chemical agents were obtained from either FUJIFILM Wako Pure Chemical Corp. (Osaka, Japan) or Sigma-Aldrich Corp. (St. Louis, MO, USA).

### 2.2. Selection of Whole Grain Wheat Samples

To compare T3 contents, five different commercially-available non-mixed breeds of whole grain wheats, including cake flour (Kitahonami), semi-hard wheat flour (Usuyume, Sumurera and Haruyutaka), bread flour (Haruyokoi) and bran (consisting of the epidermis and germ) were purchased from a market. 

### 2.3. Measurement of Vitamin E Content

V.E (α-, β-, γ-, δ-tocopherol (Toc), α-, β-, γ-, δ-T3) concentrations were measured using high performance liquid chromatography with electrochemical detection (HPLC-ECD) as described previously with some modifications [23]. Brain regions (cerebral cortex, cerebellum and hippocampus), serum, liver, various wheat samples, and crushed bran were individually mixed with 1% NaCl solution (0.5 mL), 6% pyrogallol solution (2 mL), and 35% KOH solution (2 mL), and 2,2,5,7,8-pentamethyl-6-chromanol (PMC) was used as an internal standard. The mixture was saponified at 100 °C for 45 min. After cooling, 1% NaCl solution and a mixture of hexane-ethyl acetate (9:1, by vol.) were added. After mixing, the extracts were evaporated under nitrogen gas, and methanol (0.15 mL) was added to the residue. The solutions were analyzed by HPLC (NanoSpace SI-2; Shiseido Co., Ltd., Tokyo, Japan) using a Develosil C30-UG-3 (2.0 × 250 mm; Nomura Chemical Co., Ltd., Aichi, Japan) HPLC column. The mobile phase consisted of a mixture of HPLC-grade methanol and H_2_O (97:3, by vol.) including 0.7% NaClO_4_·H_2_O. The peaks of each VE isoform were analyzed using SMC-21 software (Shiseido Co., Ltd.).

### 2.4. Total Serum Cholesterol Content

Total serum cholesterol content was measured using a commercial kit (#437-17501; Cholesterol E-test Wako), according to the manufacturer’s protocol. Cholesterol is oxidized by cholesterol oxidase to produce hydrogen peroxide. *N*-Ethyl-*N*-(2-hydroxy-3-sulfopropyl)-3,5-dimethoxyaniline, sodium salt, H_2_O_2_, and 4-aminoantipyrine undergo oxidative condensation in the presence of peroxidase, which results in the production of a blue dye that can be measured by monitoring absorbance at 600 nm using a spectrophotometer.

### 2.5. Measurement of Antioxidative Enzyme Activities

The quantification of superoxide dismutase (SOD) activity was performed using a SOD determination kit (#S311; Dojindo Laboratories, Kumamoto, Japan) according to the manufacturer’s protocol. This method is based on the xanthine oxidase reaction, which induces superoxide production. Water-soluble tetrazolium salts (WST: 5-[2,4-Bis(sodiooxysulfonyl)phenyl]-2-(4-nitrophenyl)-3-(4-iodophenyl)-2H-tetrazole-3-ium)-1 reacts with superoxide and is reduced to WST-1 formazan. In the presence of SOD, however, superoxide reacts with SOD and produces hydrogen peroxide, with a consequent decline in WST-1 formazan generation. In this assay, SOD activity is expressed as the ratio of decreased WST-1 formazan, and was determined at 450 nm absorbance using a microplate reader (#51119300. Multiskan GO; Thermo Fisher Scientific Inc., Hercules, CA, USA).

Glutathione peroxidase (GPx) activity was determined by monitoring β-nicotinamide adenine dinucleotide phosphate (NADPH) levels using a commercial kit (glutathione peroxidase cellular activity assay kit; Sigma-Aldrich), according to the manufacturer’s protocol. Samples were mixed with reduced glutathione (GSH), glutathione reductase (GR), and NADPH, and then rapidly incubated with *tert*-butyl hydrogen peroxide. Hydrogen peroxide is reduced by GPx, while GSH is oxidized to glutathione (GSSG). GR reduces GSSG to GSH and concomitantly oxidizes NADPH to NADP^+^. Consequent reductions in NADPH levels were measured at 340 nm absorbance every 10 s for 1 min. SOD and GPx activities were expressed per mg protein in the samples. Catalase (CAT) activity can be determined by monitoring hydrogen peroxide (H_2_O_2_) levels. Sample homogenates were mixed with 5 mM K-phosphate buffer, which was then rapidly incubated with hydrogen peroxide. Hydrogen peroxide is reduced by CAT. The levels of H_2_O_2_ were measured by monitoring absorbance at 240 nm.

### 2.6. Western Blotting

All samples were sonicated in lysis buffer and applied to western blotting as described previously with some modifications [17]. The lysates were centrifuged (15,000 rpm, 20 min) and the protein content of the supernatant was determined using a Bio-Rad protein assay (#500-006JA; Bio-Rad Japan, Tokyo, Japan) according to the manufacturer’s procedure. Protein extracts (20 μg) were separated on 12% SDS-polyacrylamide gels and transferred to nitrocellulose membranes (Merck KGaA, Darmstadt, Germany). The transferred membranes were stained with Ponceau S solution (Sigma-Aldrich) for 5 min and washed with 1% acetic acid. Images of the membranes were obtained (LAS-3000 system; GE Healthcare UK Ltd., Buckinghamshire, England). Ponceau S staining was used to normalize protein expression. The membranes were washed in TBS [Tris-HCl-buffered saline, pH 7.6] containing 0.5% Tween-20 (TBS-T) and incubated in blocking solution (TBS-T containing 1% non-fat milk) for 1 h at room temperature (R/T). The membranes were washed in TBS-T, and then treated with rabbit anti-SOD1 polyclonal antibody (#bs-10216R; Bioss Inc., Wobun, MA, USA), rabbit anti-GPX1 polyclonal antibody (#bs-3882R; Bioss Inc.) at 1:500 or rabbit anti-catalase polyclonal antibody (#ab195306; Abcam Plc., Cambridge, UK) at 1:2000, mammalian target of rapamycin (mTOR) (#2972; Cell Signaling Technology (CST) Inc., Danvers, MA, USA) at 1:1000, phospho-mTOR (Ser2448, #2971; CST Inc.) at 1:500, Protein kinase B (Akt) (#9272; CST Inc.) at 1:4000, and phospho-Akt (Ser473, #4060; CST Inc.) at 1:1000, dilution overnight at 4 °C. Anti-rabbit IgG HRP antibody (Promega Corp., Madison, WI, USA) was used as a secondary antibody at 1:4000 dilution for 1 h at R/T. All western blotting experiments were performed at least three times. All chemiluminescent signals were generated by incubation with the detection reagents (Immobilon Western Chemiluminescent HRP substrate; Merck) according to the manufacturer’s procedure. To confirm the western blot procedure, the membranes were reprobed with anti-α tubulin antibody (#2125; CST Inc.). The relative intensities were determined using the LAS-3000 system. The intensities of Ponceau S, SOD1, GPx1, CAT, mTOR, p-mTOR, Akt, p-Akt were quantified using ImageJ software (ImageJ 1.48v; National Institutes of Health, Bethesda, MD, USA). Expression ratios were calculated by dividing the SOD1, GPx1 and CAT density values by those of Ponceau S. Relative expressions of p-mTOR and p-Akt were calculated by dividing by the expressions of mTOR and Akt.

### 2.7. Analysis of Lipid Peroxidation

To analyze lipid oxidation, we measured thiobarbituric acid reactive substances (TBARS). Lipid peroxides were measured using Yagi’s method, as described previously with some modifications [24]. An aliquot (50 μL) of the sample homogenates was mixed with 100 μL of 5 mM EDTA, 2 mL of 1% phosphoric acid, and 1 mL of 0.7% thiobarbituric acid. The mixture was heated to 100 °C in a block heater for 45 min. After cooling on ice, the sample was incubated with 2 mL of butanol for 3 min. Following centrifugation at 3000 rpm for 10 min at 4 °C, the upper layer was isolated and absorbance at 535 nm was measured using a spectrophotometer (UV-1200; Shimadzu Corp., Kyoto, Japan). This marker of oxidative stress was expressed per mg protein in the samples.

### 2.8. Statistical Analysis

Data were plotted as means ± SE, and were analyzed using the test of rejection of Smirnoff-Grubbs. After that data were analyzed using Tukey-Kramer’s method or a two-way analysis of variance; * *p* < 0.05, ** *p* < 0.01 and *** *p* < 0.001.

## 3. Results

### 3.1. Determination of HPLC Condition

**Figure 1:**
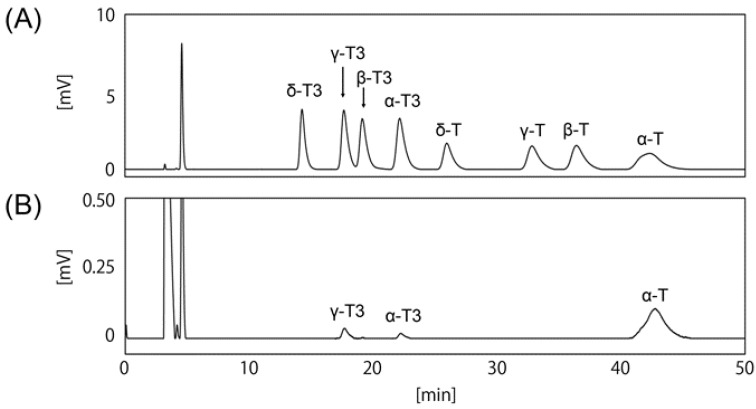
Determination of vitamin E (VE) isoforms levels using HPLC-ECD. The peaks show the 8 pure VE isoforms (**A**). The peaks show each VE isoform isolated from the cerebral cortex of normal young mouse brain (**B**) (3-month-old).

To determine the anti-obesity effect of T3s, it was necessary to measure the content of T3s in each food component. As shown in Figure 1A, the mixed-8 standard VE isoforms were completely separated by HPLC-ECD. Next, we investigated the ability to detect T3s in the mouse tissue samples, and confirmed the presence of α-Toc and α- and γ-T3 in the normal mouse brain (Figure 1B). However, the peak levels of α- and γ-T3 were very low compared to α-Toc.

### 3.2. Measurement of Vitamin E Contents of Whole Grain Wheats, Bran and Control Diet

**Figure 2:**
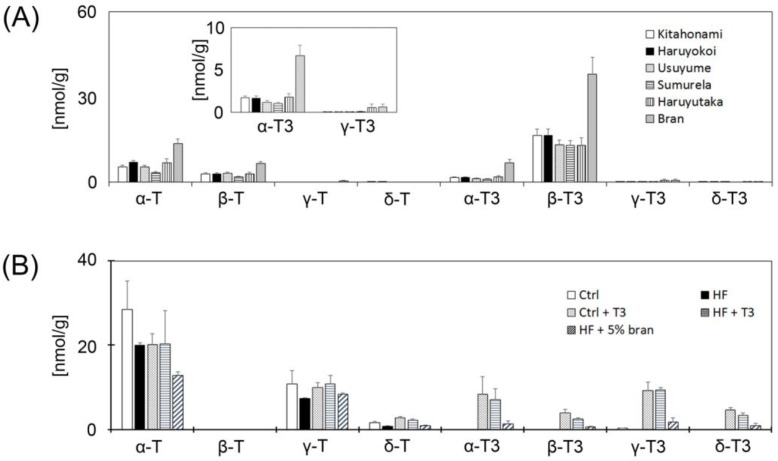
Measurement of vitamin E (VE) contents in food components. Five different kinds of pure whole grain wheat samples and a bran sample were subjected to measurement of VE levels (**A**). Individual VE contents of the five different diets (**B**). Each column represents the mean of four independent experiments. Ctrl = Control, HF = High-fat, Ctrl + T3 = Control + Tocotrienols, HF + T3 = High-fat + Tocotrienols, HF + 5% Bra*n* = High-Fat + 5% Bran.

To compare T3 levels in whole grain wheat samples and bran, we purchased five different kinds of pure whole grain wheat samples and bran. As shown in Figure 2A, all samples contained α-Toc and α-T3. Bran showed the highest levels of α-Toc and α-T3 of all the whole grain wheat samples; therefore, we used bran in subsequent experiments (Figure 2A). Surprisingly, of the VE isoforms, the content of β-T3 was the highest in all samples. Next, we measured the VE contents of the five different mouse diets. As shown in Figure 2B, the α-Toc content was relatively high compared to the other VE isoforms. T3 contents of both the T3 supplemented groups (Ctrl + T3 and HF + T3) were higher than the other non-T3 supplemented diet groups. The T3s content of the 5% bran supplemented HF group was higher than those of the control and HF groups.

### 3.3. T3s and Bran Inhibited Body Weight Gain in High-Fat Diet-Treated Mice

**Figure 3:**
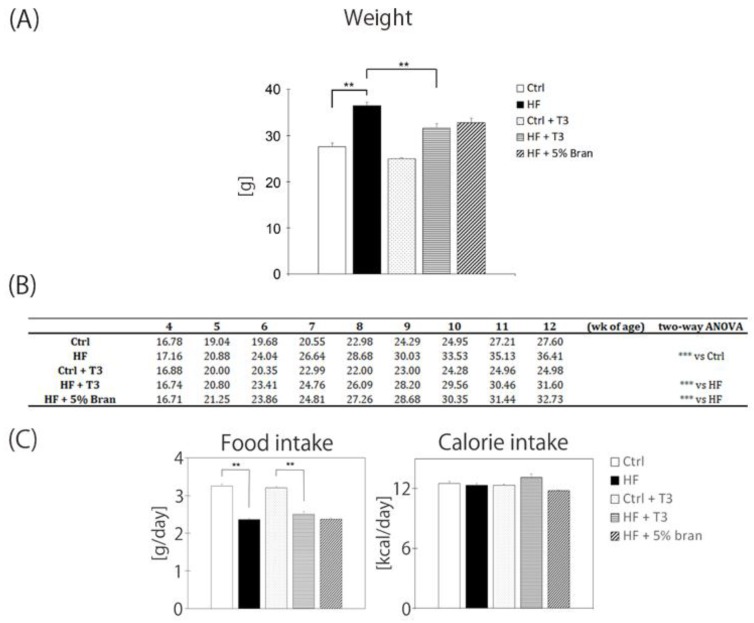
The effects of tocotrienols and bran on weight gain in mice. Changes in the average weight of each mouse group at 12 weeks of age (**A**). Changes in the average weight of each mouse group from 4 to 12 weeks of age (**B**). Food and calorie intakes for each mouse group (**C**). Control (Ctrl, *n* = 8), high fat (HF, *n* = 7), control + T3s (Ctrl + T3, *n* = 8), HF + T3 (*n* = 8), HF + 5% bran (*n* = 8). Data were analyzed using Tukey-Kramer’s method (**A**,**C**) and a two-way analysis of variance (**B**). ** *p* < 0.01 and *** *p* < 0.001.

Mice were fed five different diets from 4 to 12 weeks of age. The body weight of all mice gradually increased in a time-dependent manner (Figure 3B). Treatment with the HF diet significantly increased body weight, and co-treatment with T3s significantly inhibited the body weight gain (Figure 3A). There were significant interactions of body weight gain between the control vs HF, HF vs HF + T3, and HF vs HF + 5% bran for the 8-week feeding period (Figure 3B). The amount of food intake of the two HF diet-treated groups was significantly lower than the other two control diet-treated groups in the presence or absence of T3. However, the calorie intake did not significantly differ among the groups (Figure 3C).

### 3.4. T3s and Bran Significantly Decreased Serum Cholesterol Levels

**Figure 4:**
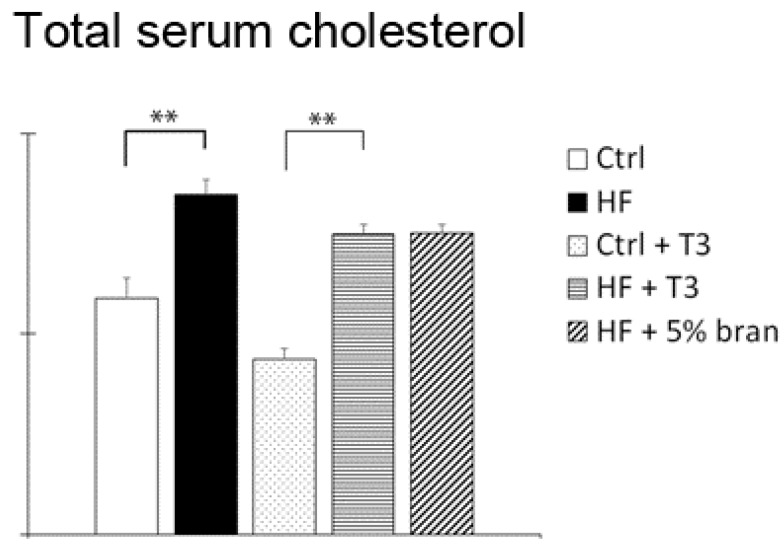
Treatment with tocotrienols and bran did not decrease serum cholesterol levels in high-fat diet-treated mice. Control (Ctrl, *n* = 8), high fat (HF, *n* = 7), control + T3s (Ctrl + T3, *n* = 8), HF + T3 (*n* = 8), HF + 5% bran (*n* = 8). The data was analyzed using Tukey-Kramer’s method. ** *p* < 0.01

We measured total serum cholesterol levels using a commercial kit. As shown in Figure 4, the cholesterol level of the HF group was significantly higher than that of the control. Co-treatment with T3s or 5% bran tended to decrease the serum cholesterol level in the HF group. However, there were no significant differences in the serum cholesterol level in the presence or absence of T3s.

### 3.5. Vitamin E Contents of Mouse Brains Did Not Significantly Differ between Diet Groups

**Figure 5:**
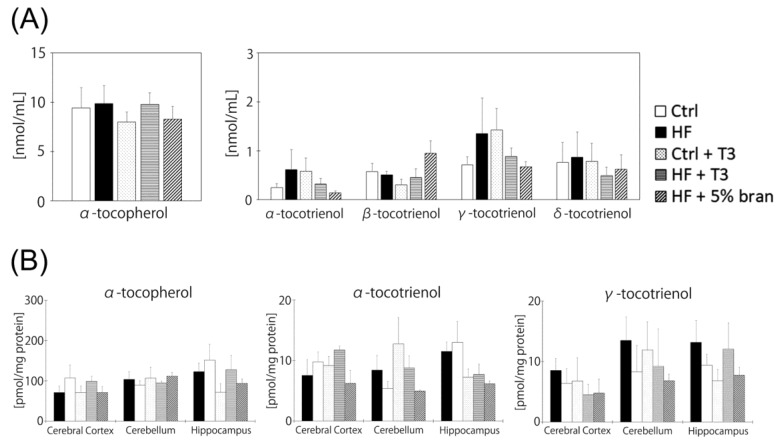
Measurement of vitamin E contents in serum (**A**) and cerebral cortex, cerebellum and hippocampus (**B**). Control (Ctrl, *n* = 8), high fat (HF, *n* = 7), control + T3s (Ctrl + T3, *n* = 8), HF + T3 (*n* = 8), HF + 5% bran (*n* = 8). The data was analyzed using Tukey-Kramer’s method.

To clarify the mechanism of the inhibitory effect of T3s and bran on body weight gain and serum cholesterol levels, we measured serum VE contents. Unexpectedly, VE contents did not significantly differ between the diet groups (Figure 5A). We also measured VE contents of brain regions in mice. Although we could detect α- and γ-T3, there were no significant differences in VE contents in brain regions in the diet groups (Figure 5B). Beta- and δ-T3 were not detected in the brains of all diet groups.

### 3.6. Changes in Antioxidant Enzyme Activities and Protein Expressions in the Mouse Brain

**Figure 6:**
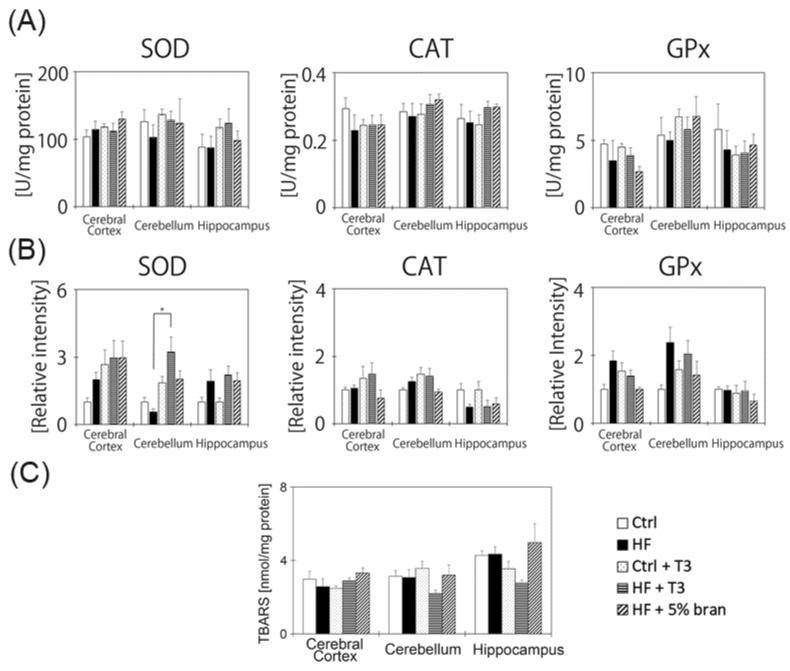
The effects of tocotrienols and bran on the antioxidant network in the mouse brain. Activities of the antioxidant enzymes super oxide dismutase (SOD), catalase (CAT) and glutathione peroxidase (GPx) were measured (**A**). Relative intensities of antioxidant enzyme protein expressions were measured by western blotting (**B**). Data are expressed as the ratio of each antioxidative protein band intensity relative to that of Ponceau S. The ratio in 3-month-old normal mice in each region was set to 1. Lipid peroxidation levels in each mouse group were measured as TBARS (**C**). Control (Ctrl, *n* = 8), high-fat (HF, *n* = 7), control + T3s (Ctrl + T3, *n* = 8), HF + T3 (*n* = 8), HF + 5% bran (*n* = 8). A minimum of three wells was used per experiment. Each column represents the mean of three independent experiments. The data was analyzed using Tukey-Kramer’s method; * *p* < 0.05. Details of the sample preparation and experimental conditions are described in the Materials and Methods.

Although treatment with T3s and bran inhibited body weight gain in HF diet-treated mice, the amounts of T3s in the different brain regions of mice did not change. To clarify the relationship between HF diet and brain oxidative injury, we measured antioxidant enzyme activities and protein expressions, such as SOD, CAT and GPx. However, for enzyme activities, there were no significant differences among all groups (Figure 6A). For protein expression, there were no significant differences among all groups, except for SOD in the cerebellum (Figure 6B). Additionally, we measured TBARS contents in all samples, and there were no significant differences among all groups (Figure 6C).

## 4. Discussion

### 4.1. Bran Contains High Levels of T3s and Attenuates Body Weight Gain

To prevent HF diet-induced obesity and its related brain antioxidant network dysfunction, we treated control and HF diet fed mice with T3s. Furthermore, to clarify the preventative effect of natural foods, we fed bran, which contains the epidermis and germ, to the HF diet group. In our study, bran was high in α- and γ-T3 compared to the 5 other whole grain wheat samples (Figure 2A). It is well known that rice bran and barley germ are also high in T3s [25]. Prior to initiating the present study, we confirmed the VE contents of all five foods before and after heat-drying. There were no significant differences in VE contents in each food before and after heat-drying (0, 15, 30, 45 and 60 min heat drying), and thus each food was heated for 30 min for 100 °C. To prevent oxidation, food samples were stored in a fridge in an air-sealed, light-shielded container, and small portions of the foods were provided to the animal cages. Since all whole grain wheat, bran and control pellets were made from natural plants including corn, soy flour and other cereals, the various mouse diets contained a relatively high β-T3 content.

Treatment with 5% bran or T3s significantly decreased the body weight gain in HF-treated mice (Figure 2). Bran contains many other antioxidants, phytochemicals and fiber [25,26], raising the possibility that other factors played a role in inhibiting body weight gain. However, treatment with T3s significantly decreased the body weight gain in HF-treated mice. These results indicate that one of the causes of the attenuation of body weight gain was the effect of T3s.

Opinions about the anti-obesity effect of T3s differ among researchers. Zhao et al. reported [20] that treatment with γ-T3 in one-month-old mice for 4 weeks attenuated HF-induced body weight gain via decreased plasma glucose and insulin levels. Wong et al. reported [21] that treatment with T3s from palm oil reduced atherosclerotic lesions via decreases in blood glucose levels and restores insulin imbalance tolerance. The HMG-CoA inhibitory [18] and cholesterol-lowering effects [27] of T3s are well known, and these studies are consistent with our data. In the present study, we used young mice (one-month-old); thus, from another view point, treatment with T3s may attenuate body growth in mice.

### 4.2. Tocotrienols Do Not Reach the Brain or Affect the Brain Antioxidant Network

**Figure 7:**
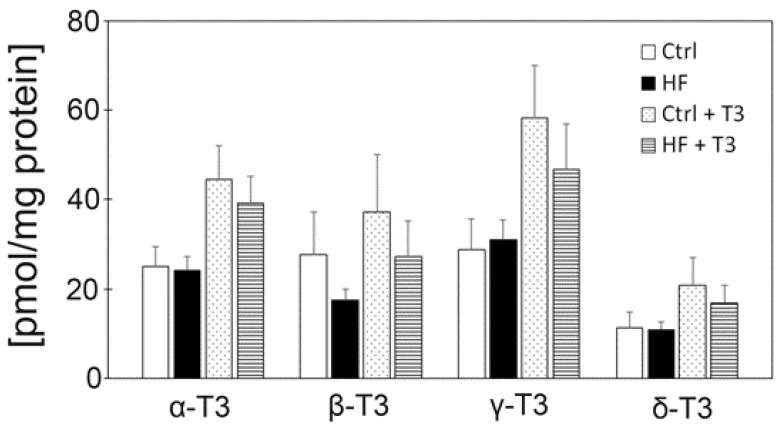
Measurement of T3 contents in liver. Control (Ctrl, *n* = 8), high fat (HF, *n* = 8), control + T3s (Ctrl + T3, *n* = 8), HF + T3 (*n* = 8), HF + 5% bran (*n* = 7). The data was analyzed using Tukey-Kramer’s method. Experimental and animal conditions were same to the other experiments.

T3s have neuroprotective effects in vivo [28] and in vitro [16,17]. To clarify the effects of T3s on the brains of HF-treated mice, we measured VE contents in the serum and different brain regions. We detected α- and γ-T3s in all samples, and the levels of VE did not significantly differ between all samples (Figure 5). Previously, we confirmed that α- and γ-T3 levels tended to increase in the livers of T3s-treated mice (Figure 7). These results and our previous data indicate that T3s may not reach the brain. However, VE has been shown to improve liver function in conditions such as non-alcoholic steatohepatitis [29]. Thus, the anti-obesity effect of T3s may be related to improvements in liver function.

Antioxidant enzyme activities and the TBARS assay revealed no significant differences among samples, except in certain regions of the brain. In HF diet-treated groups, treatment with T3s significantly increased SOD protein expression in the cerebellum compared to the untreated group. On the other hand, no significant differences in the cerebral cortex and hippocampus were observed among all groups (Figure 6B). However, SOD and GPx protein expressions in the cerebral cortex tended to increase in the HF-treated group compared to the controls. Furthermore, SOD protein expression in the hippocampus also tended to increase in the HF-treated group compared to the control. These results indicated that the production of oxidative stress was slightly increased in the brain by HF diet feeding. However, the ratio of oxidative stress production by the HF diet was small. Finally, we determined p-mTOR and pAkt protein expressions, as these proteins are upstream in the signaling pathway of the oxidative response in living tissues, and are related to autophagy (Supplemental Figure S1). However, there were no significant differences among the samples.

### 4.3. Relationship between Brain Oxidation and T3 Levels

In our experimental models, T3s reached the liver but not the serum or brain. However, T3 treatment in normal aged rats significantly improved cognitive function [28]. In the previous study, treatment with T3s in young control rats did not change cognition. Because the levels of oxidative products were high in the brains of normal aged rats and mice, we hypothesized that changes in brain T3s levels may be related to the level of brain oxidation. A factor that may be relevant to this mechanism is the role of blood brain barrier dysfunction. We previously reported that normal-aged rat brains showed impaired tight junctions [28]. The protein expressions, such as claudin-5, occludin and junctional adhesion molecule-1 were significantly decreased in normal-aged and young hyperoxia rat brains. The collapse of tight junctions may accelerate the influx of T3s to the brain. Furthermore, treatment with T3s to normal-aged rats improved learning ability in the Morris water maze test, and significantly increased brain α- and γ-T3 levels compared to the untreated groups. To clarify the responsible mechanism, we are continuing to study this phenomenon using human umbilical vein endothelial cells (HUVEC) cells.

## 5. Conclusions

In this study, we demonstrated the anti-obesity effects of T3s and bran. However, the relationship between HF diet-induced brain oxidation and the neuroprotective effects of T3s remains unclear. Obesity is a high-risk factor for serious severe diseases including neurodegenerative disorders. In the near future, we aim to demonstrate the role of T3s in the anti-obesity effect and in the maintenance of brain function.

## Supplementary Materials

The following are available online at https://www.mdpi.com/2072-6643/11/4/830/s1, Figure S1: Relative intensities of p-mTOR (A) and p-Akt (B) protein expressions were measured by western blotting. Protein expression was expressed relative to the non-phosphorylated band. The ratio in 3-month-old normal mice in each region was set to 1. The data was analyzed using Tukey-Kramer’s method. Table S1: Composition of diets.
