# Supplementary figures and images for: Anti-Obesity Effects of Tocotrienols and Bran in High-Fat Diet-Treated Mice
Source: Nutrients. 2019 Apr 12;11(4):830. doi: 10.3390/nu11040830 (PMC6521046; doi:10.3390/nu11040830)

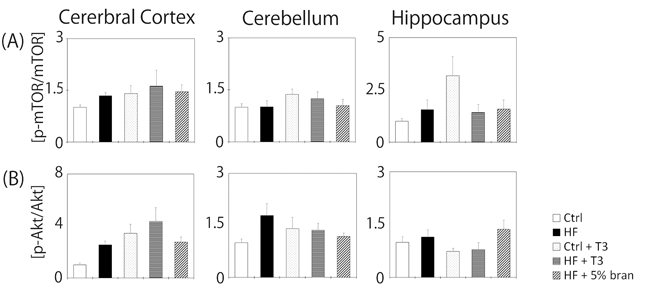

Supplement: Supplementary file 1 [file nutrients-11-00830-s001.zip › Supple Fig and Table/Supplemental Figure 1.tif]

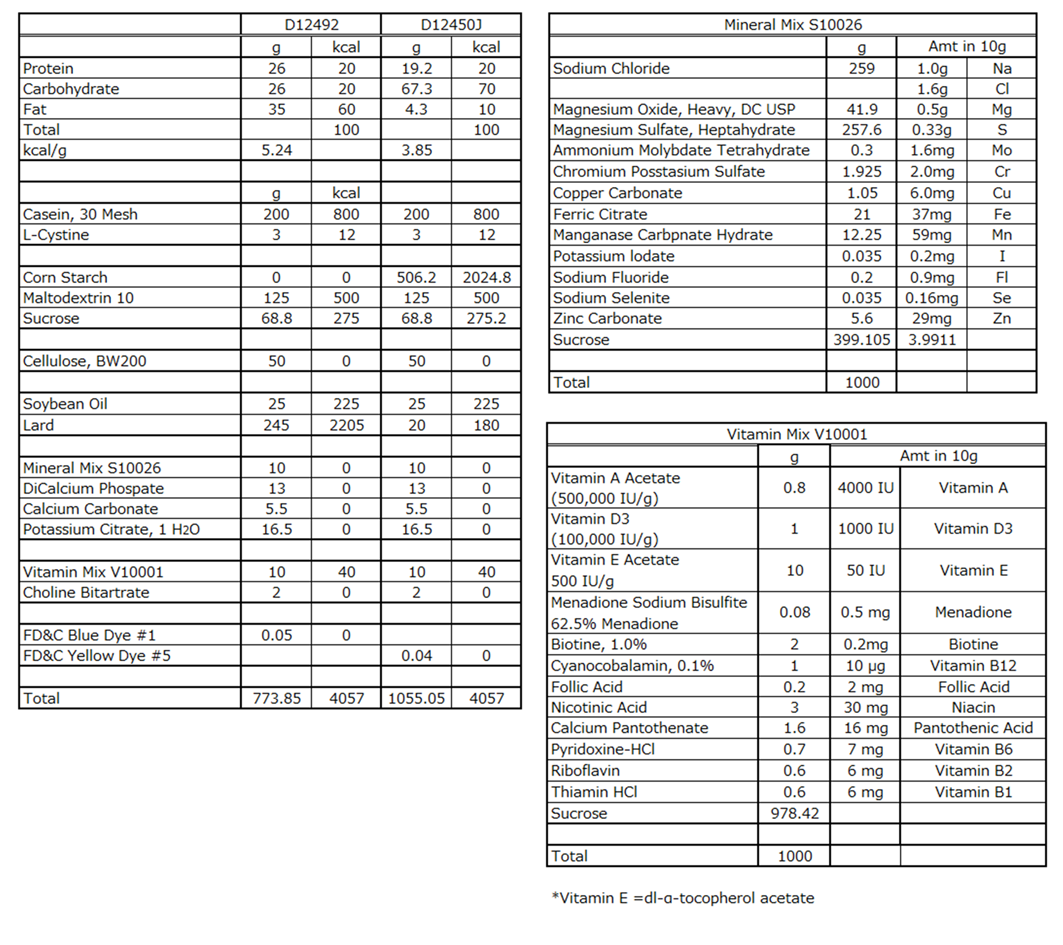

Supplement: Supplementary file 1 [file nutrients-11-00830-s001.zip › Supple Fig and Table/Supplemental Table 1.tif]
